# Pancreatic autoantibodies and CD14^+^CD16^+^ monocytes subset are associated with the impairment of ß-cell function after simultaneous pancreas-kidney transplantation

**DOI:** 10.1371/journal.pone.0212547

**Published:** 2019-02-22

**Authors:** Cristian Rodelo-Haad, Maria Luisa Agüera, Andres Carmona, Maria Dolores Navarro, Julia Carracedo, Alberto Rodriguez-Benot, Pedro Aljama

**Affiliations:** 1 Maimonides Biomedical Research Institute of Cordoba (IMIBIC)/Reina Sofia University Hospital/University of Cordoba, Cordoba, Spain; 2 Nephrology Unit. Reina Sofia University Hospital, Cordoba, Spain; 3 RETICs Red Renal (Instituto de Salud Carlos III), Madrid, Spain; 4 Departamento de Genética, Fisiología y Microbiología, Facultad de Biología, Universidad Complutense de Madrid, Instituto de Investigación Sanitaria Hospital 12 de Octubre (imas12), Madrid, Spain; Université Paris Descartes, FRANCE

## Abstract

Pancreatic autoantibodies (AAb) has been associated with a worse pancreas graft survival after simultaneous pancreas-kidney transplantation (SPK). However, due to the variable time for AAb to become positive and the lack of early biomarkers suggesting such autoimmune activation, the mechanisms leading ß-cell destruction remain uncertain. The present study aimed to evaluate the association between post-transplant AAb and the functional impairment of the pancreatic ß-cell and also the association of such AAb with inflammation after SPK. In a longitudinal study, we analyzed the impact of post-transplant glutamic acid decarboxylase (GAD-65) and the insulinoma-associated autoantigen 2 (IA-2) AAb on pancreas graft function. Serum Hb1Ac and C-peptide (C-pep) were longitudinally compared between a group with positive posttransplant AAb (AAb+; n = 40) and another matched group with negative AAb (AAb-; n = 40) until the fifth year following seroconversion. In the cross-sectional analysis, we further evaluated the systemic signatures of inflammation by measuring pro-inflammatory CD14^+^CD16^+^ monocytes by flow-cytometry and interleukin 17-A serum levels in 38 SPK recipients and ten healthy controls. In the longitudinal study, patients with AAb+ showed higher levels of Hb1Ac (p<0.001) and lower C-pep levels (p<0.001) compared to those who remained AAb- throughout the follow-up. In the cross-sectional study, AAb+ patients showed a higher percentage of CD14^+^CD16^+^ monocytes compared with those with AAb- and the healthy controls (6.70±4.19% versus 4.0±1.84% and 3.44±0.93%; p = 0.026 and 0.009 respectively). Also, CD14^+^CD16^+^ monocytes correlated with Hb1Ac and C-pep serum levels. Multivariate logistic regression showed that posttransplant AAb+ was independently associated with a higher percentage of pro-inflammatory monocytes (adjusted-OR 1.59, 95%CI 1.05–2.40, p = 0.027). The group of patients with positive AAb also showed higher levels of IL17A as compared with the other groups (either healthy control or the negative AAb subjects). In conclusion, pancreatic AAb+ after SPK were not only associated with higher Hb1Ac and lower c-peptide serum levels but also with an increased percentage of CD14^+^CD16^+^ monocytes and higher levels of circulating IL17-A.

## Introduction

Type 1 Diabetes (T1D) is an autoimmune and inflammatory disease associated with the destruction of pancreatic insulin-producing ß-cells [1–4]. In patients with end-stage renal disease (ESRD) secondary to T1D, simultaneous pancreas and kidney transplantation (SPK) has become the best option to restore glucose control and kidney function [5–7]. Classically, T1D is developed in genetically susceptible individuals in whom precipitating events trigger inciting immune and inflammatory mechanisms [8]. A disequilibrium between effectors T-cells and T-regs may be associated with the onset of ß-cell function decline; thus, auto-reactive T-cells are determinant in this expanding autoimmune process [1,9]. In this line, the Th1 IFN*y*-producing cells and the Th17 IL-17-producing cells are closely related to autoimmunity [10]. Indeed, as the proportion of IL-17-secreting CD4^+^ has been shown to be increased in patients with T1D, its development could be prevented by neutralizing such IL-17-secreting cells [11,12]. Functionally, IL-17 modulates the traffic of different immune cells such as monocytes [10,13]. In turn, monocytes have been involved in stimulating memory T cells to secrete IL-17 and IFN-*y* [14]. Therefore, recruitment and differentiation of monocytes together with the IL-17 cytokine [15] are both implicated in the immune process preceding T1D.

In healthy subjects, the predominant monocytes subset is the CD14^++^CD16^—^. In contrast, pathologies leading to chronic inflammation induce a change in the subset of those expressing the CD14^+^CD16^+^ surface molecules that increase the production of inflammatory cytokines [16–18]. In fact, in new-onset T1D, monocytes are keen on secreting inflammatory cytokines [19].

On the other hand, the number and the type of islet autoantibodies (AAb) regulate the time to T1D onset [20,21]. Insulin, glutamic acid decarboxylase (GAD-65), and insulinoma-associated protein 2 (IA-2) are some of the identified AAb implicated in the development of T1D [20,22]. In preclinical T1D, patients with positive AAb show dysregulation of glucose even two years before the advent of cardinal T1D symptoms [23,24]. After SPK, the impact of pancreatic AAb on pancreas graft survival remains controversial [25–27]. Recent studies have demonstrated that pancreatic autoantibodies are risk factors for a worse pancreas graft survival [28,29]. However, the underlying mechanism and the timeline through which AAb address a poor pancreas graft survival is yet to be elucidated.

Since pancreatic autoantibodies have been demonstrated to be a strong predictor of T1D recurrence after SPK; we aimed to evaluate whether pancreatic AAb had a role in disturbing the long-term endocrine function of the pancreas graft. We hypothesize that one of the possible mechanisms through which such an effect is promoted is by inducing micro-inflammation.

## Materials and methods

### Patients

From the one-hundred and seventeen SPK performed at Reina Sofia University Hospital, Cordoba- Spain from January 2005 to December 2014, we first conducted a longitudinal study in patients in whom pancreatic AAb were evaluated (S1 Dataset). Only those patients with at least two serial AAb positive determinations and a follow-up of one to five years after AAb positivity were included. The AAb + population was compared with an AAb- cohort matched according to age and transplantation vintage. Pancreas graft functional parameters such as C-peptide (C-pep) and glycated hemoglobin (Hb1Ac) were evaluated at 12, 24, 48 and 60 months after the detection of AAb positive.

We further performed a cross-sectional study in a cohort of patients in whom pancreas and kidney graft function were considered within the normal range at the time of inclusion: fasting plasma glucose <126 mg/dL, fasting C-pep (>0.20 pmol/mL), non-requirement of exogenous insulin and glomerular filtration rate (GFR) >60 mL/min/1.73m^2^ estimated by MDRD-4 (S2 Dataset). In these patients, AAb status, the CD14^+^CD16^+^ monocytes subtype and IL-17A serum levels were evaluated from the same blood sample. The amount of AAb (GAD65 and IA-2) were quantified in all patients using radioimmunoassay (Cisbio Bioassays, Codolet, France) and considered positive when IA-2 AAb were above 1 U/ml and GAD-65 >1 UI/ml. Inflammatory parameters such as albumin, ferritin, and high-sensitivity C-reactive protein (*hs*-CRP) and parameters associated with pancreas graft function such as fasting glucose, C-pep by radioimmunoassay (Izotop Bioassays, Budapest, Hungary) and fasting plasma insulin were also analyzed at the time of blood drawn.

Patients with preformed HLA donor-specific antibodies (DSA+), clinical history of T-Cell mediated rejection, active malignancies, active infections or inflammation or with other autoimmune diseases different to type 1 diabetes (T1D) and those positive for hepatitis B or C virus were excluded. None of the patients were receiving insulin for glucose control or were HIV positive Clinical and demographic variables from each patient were also gathered. Ten healthy subjects, matched for gender and age, were selected as a control group for monocytes and cytokine determinations. Induction therapy was the same for all patients. It included Basiliximab [Simulect; Novartis, East Hanover, NJ, United States]), 20 mg (day 0 and day 4). Moreover, all patients analyzed received the same immunosuppressive regimen at the time of grafting, which included corticosteroids, mycophenolic acid, and calcineurin inhibitors. Corticoids (Prednisone) was maintained as long-term therapy. The study was approved by the local institutional ethics committee (Comité de Ética de la Investigación de Córdoba; ref. 2890, protocol PCL2015). Written informed consent was obtained from all patients. None of the transplant donors were from a vulnerable population, and all donors or next of kin provided written informed consent that was freely given.

### Determination of CD14 and CD16 mononuclear phenotype expression in peripheral blood and analysis of plasma interleukin-17 and Type II interferon-*y* by high sensitivity ELISA

A 10-mL sample of peripheral venous blood was drawn from patients and healthy subjects into tubes that contained lithium heparin. Blood was incubated with the mAbs M5E2 against the molecule CD14 conjugated with peridinin chlorophyll protein (PerCP) and 3G8 against the molecule CD16 conjugated with FITC. Both, antibodies and the appropriate isotype controls were provided by Becton Dickinson (San Jose, CA). Flow cytometric analysis was performed with a FACScalibur flow cytometer (Becton Dickinson). Absolute CD14^+^CD16^++^ monocyte numbers were obtained using BD TruCount Tubes (Becton Dickinson). To calculate the median fluorescence intensity (MFI) of the receptors, the flow cytometer was calibrated with BD Calibrite 3 beads (Becton Dickinson) to adjust set fluorescence compensation. Serum levels of interleukin-17 (IL-17) and Type II interferon *y* (IFN*y*) were analyzed by high sensitivity ELISA using the Human IL-17A High Sensitivity ELISA kit (eBioscience, Bender MedSystem, Vienna, Austria) and Human IFN-y High Sensitivity ELISA kit (eBioscience) in a subgroup of patients. The samples were measured using a Power Wave XS microplate reader (Biotek, VT) set to 450 nm. Patients were grouped according to whether their plasma levels of IL-17A were higher or lower the reference values for the healthy population (reference value <0.02 pg/ml).

### Statistical analyses

Categorical variables were described using percentage and quantitative variables depending on if they followed a normal distribution (mean ± standard deviation [SD]) or not (median, interquartile range). Non-parametric data were analyzed by the Mann–Whitney U-test or Kruskal-Wallis test. The correlation between CD14^+^CD16^+^ monocytes and other variables was calculated by the Pearson correlation coefficient method. Multivariate logistic regression was used to evaluate the association between different factors and post-transplant pancreatic autoantibodies positivity; we first performed a univariate analysis, and then the multivariate model was constructed including significant variables and those that may be related to autoantibody positivity. Goodness-of-fit (calibration and discrimination ability) was performed using the Hosmer-Lemeshow test and the ROC curve. The area under the curve (AUC), sensitivity, specificity, positive and negative predictive values and the accuracy of the model were also assessed. For all tests, statistical significance was assumed at P < 0.05. Data were analyzed using SPSS Statistics software version 15.0 (SPSS, Inc., Chicago, Ill, United States) or the GraphPad Prism 6.0c (GraphPad Software, La Jolla, CA).

## Results

### Patient population, pancreatic AAb and pancreas graft functionality

A total of eighty patients were included in the longitudinal analysis. Among AAb+ patients, 40% (n = 16) were positive for GAD65 whilst 45% (n = 18) were positive for IA-2 and 15% (n = 6) were positive for both AAb. Median time from transplantation to AAb positivity was 28.7 months (IQR 4.9–55.2) which was comparable between the different types of AAb [31.2 (19.1–55.4) months for GAD65 AAb, 23.2 (3.1–55.4) for IA-2 AAb and 27.8 (5.4–59.5) months for those positive for both AAb (p = 0.92).

**Fig 1:**
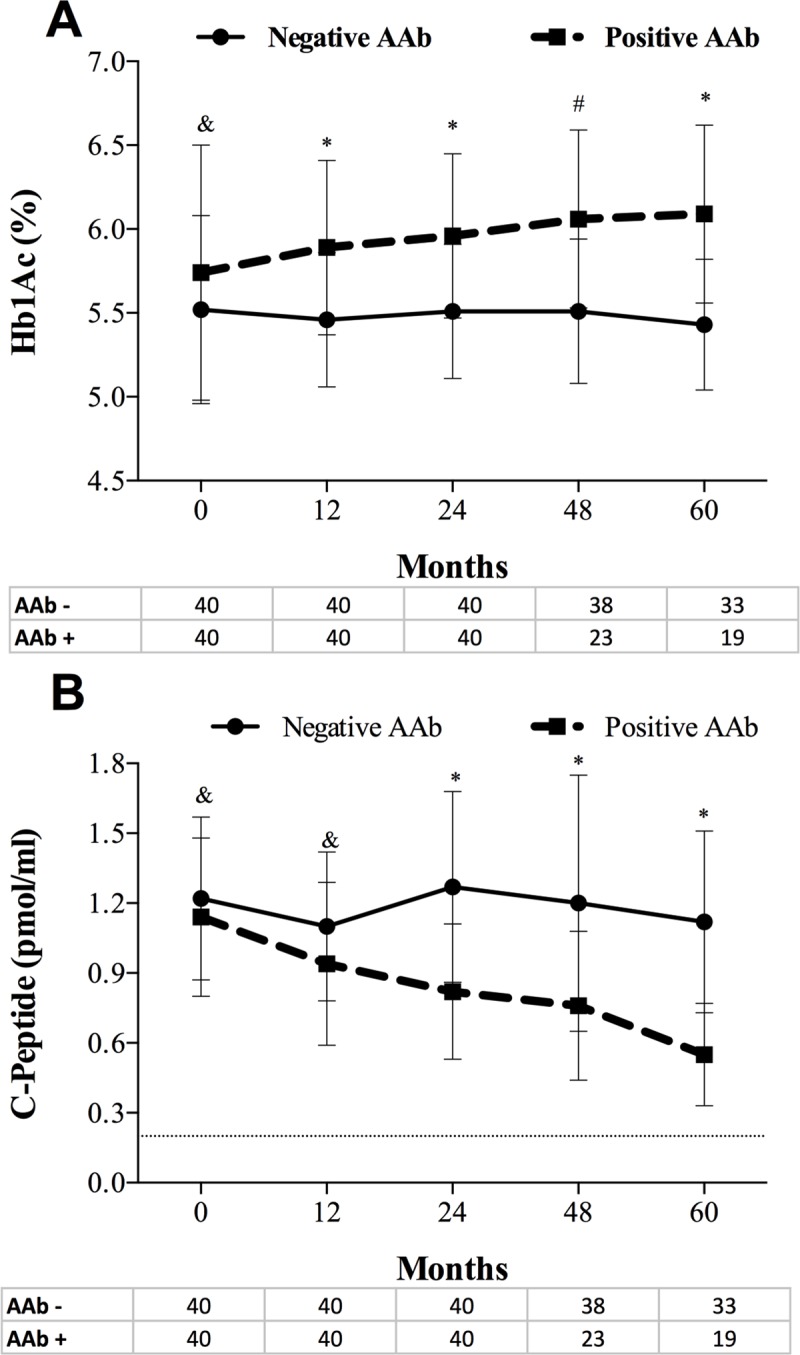
**Glycated hemoglobin (A) and C-pep (B) serum levels among patients with positive and negative pancreatic AAb.** The dark-dashed line represents positive AAb patients, and the continuous line represents AAb negative patients. **I** bar represents standard deviation. The number of patients at each time-point is depicted under x-axis. & p>0.05, * p<0.001, ^#^ p<0.01.

Compared to AAb- patients, the AAb+ patients showed higher levels of Hb1Ac (Fig 1A) and lower C-pep serum levels (Fig 1B) at 12, 24, 48 and 60 months of follow-up. Regarding differences between the different AAb subtypes, Patients positive for GAD65, IA-2, or GAD65/IA-2 had higher levels of Hb1Ac levels and lower levels of C-pep than AAb- patients at any time point after the first year of follow-up compared to AAb- patients (S1A and S1B Fig).

### Analysis of the CD14^+^CD16^+^ monocytes in healthy and transplanted subjects

To investigate whether AAb positivity was associated with an increase inCD14^+^CD16^+^ monocytes, we selected 38 patients from the previous cohort in whom inclusion criteria were fulfilled. Clinical and biochemical characteristics of patients are shown in Table 1. At the time of inclusion, all patients included were on Tacrolimus as calcineurin-inhibitor at a mean dose of 3.5 mg/day and blood trough levels of 7,5 ng/ml (IQR 4.9 to 12). Thirty-one of the patients (81.5%) were on mycophenolic acid, and all patients were on low dose prednisone (5 or 10 mg per day).

**Table 1:** Demographic characteristics of patients included in the cross-sectional study (n = 38).

Recipients		
**Age (years)** *		35.8 ± 6.2
**Gender (Male, n, %)**		28 (73.6)
**HLA class II alleles***DR3 (n*, *%)DR4 (n*, *%)R3/DR4 (n*, *%)*		10 (26.3)9 (23.7)10 (26.3)
**Induction Therapy Basiliximab (n, %)**		38 (100%)
**Transplantation Vintage (Months)** ^**a**^^,^ ^**¶**^		51.2 (23.6–83.3)
**Fasting Plasma Glucose (mmol/L)** *		5.20 ± 1.83
**Hb1Ac (%)** ^**b**^^,^ *		5.58 ± 0.55
**C-peptide (pmol/ml)** *		0.79 ± 0.29
**Albumin (g/l)** *		4.27 ± 0.33
**Ferritin (ng/ml)**		38 (15.7–73.5)
***hs*-CRP (mg/l)** ^**c**^^,^ ^**¶**^		2.1 (0.77–4.0)
**GFR by MDRD-4 (mil/min/1.73 m**^**2**^**)** ^**d**^^,^ ^**¶**^		81.0 (65.3–90.2)
**Corticosteroids dose (mg per day)**		5.66 ± 1.71
**Tacrolimus levels (ng/ml)** ^**e**^^,^ *		7.86 ± 1.78

* Mean ± Standard Deviation.

^¶^ Median—Interquartile range^.^

^a^ Transplantation Vintage, Time since transplantation

^b^ Glycated Hemoglobin

^c^ *hs*-CRP, C-Reactive Protein

^d^ GFR, Glomerular filtration rate

^e^ Tacrolimus, Tacrolimus trough serum levels.

In this cohort, 55.3% (n = 21) of the patients were AAb+ and 44.7% (n = 17) were negative for any type of autoantibody. Among AAb+, 47.6% (n = 10) were positive for GAD-65 AAb [median serum levels 4.5 (IQR 2.8–93.3 UI/ml)], 38.1% (n = 8) were positive for the IA-2 autoantibody [median serum levels 2.30 (IQR 1.20–5.51 U/ml) and 14.3% (n = 3) were positive for both GAD-65 and IA-2 (GAD-65/IA-2). There were no differences in age (p = 0.93), transplantation vintage (p = 0.50), *hs*-CRP (p = 0.93) and glomerular filtration rate (p = 0.39) in patients AAb+ vs. AAb-. Tacrolimus trough levels in the AAb+ group were 8.0 ± 1.87 pg/ml compared with 7.68 ± 1.70 pg/ml in AAB- patients (p = 0.75).

**Fig 2:**
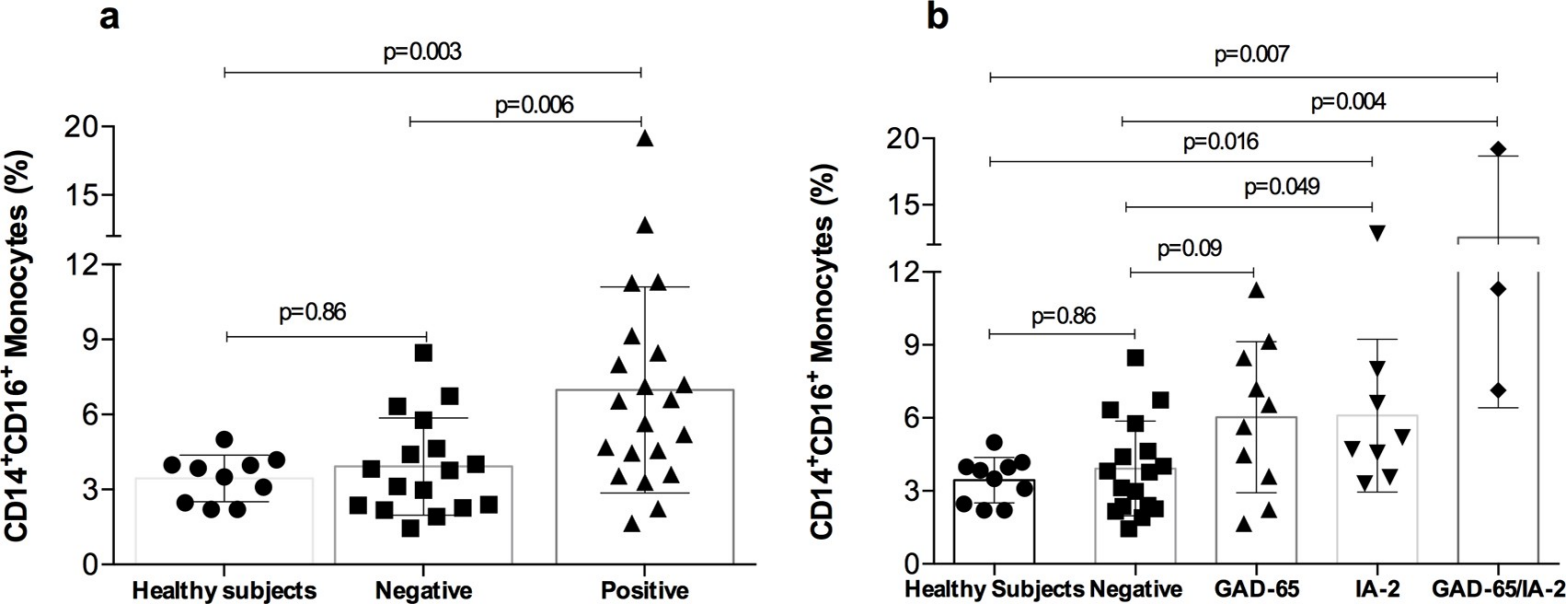
**Distribution of CD14**^**+**^**CD16**^**+**^ **monocytes between the healthy controls and those with negative or positive AAb (A) and among different AAb types (B). I** bar represents standard deviation.

The percentage of pro-inflammatory monocytes (CD14^+^CD16^+^) was higher AAb+ than AAb- patients (6.98 ± 4.11% versus 3.91 ± 1.94%, p = 0.006) and the healthy controls (6.98 ± 4.11% versus 3.44 ± 0.93%, p = 0.003; Fig 2A). By contrast, the pro-inflammatory monocytes in AAb- patients and the control group were comparable (p = 0.86, Fig 2A). Regarding differences between the different type of AAb, patients positive for GAD-65 AAb showed a moderate increase in pro-inflammatory monocytes that did not reach statistical differences as compared with AAb- patients (p = 0.083, Fig 2B). Conversely, IA-2 positive AAb patients showed a higher proportion of pro-inflammatory monocytes than AAb- patients and the healthy controls (p = 0.049 and p = 0.016 respectively). Similarly, those patients that were positive for both AAb GAD65/IA2+) had a higher proportion of CD14^+^CD16^+^ monocytes than controls (p = 0.007) and AAb- patients (p = 0.004, Fig 2B). No correlation was observed between the percentage of CD14^+^CD16^+^ and the serum levels of any of the AAb.

### CD14^+^CD16^+^ monocytes and the impairment of glucose control

**Fig 3:**
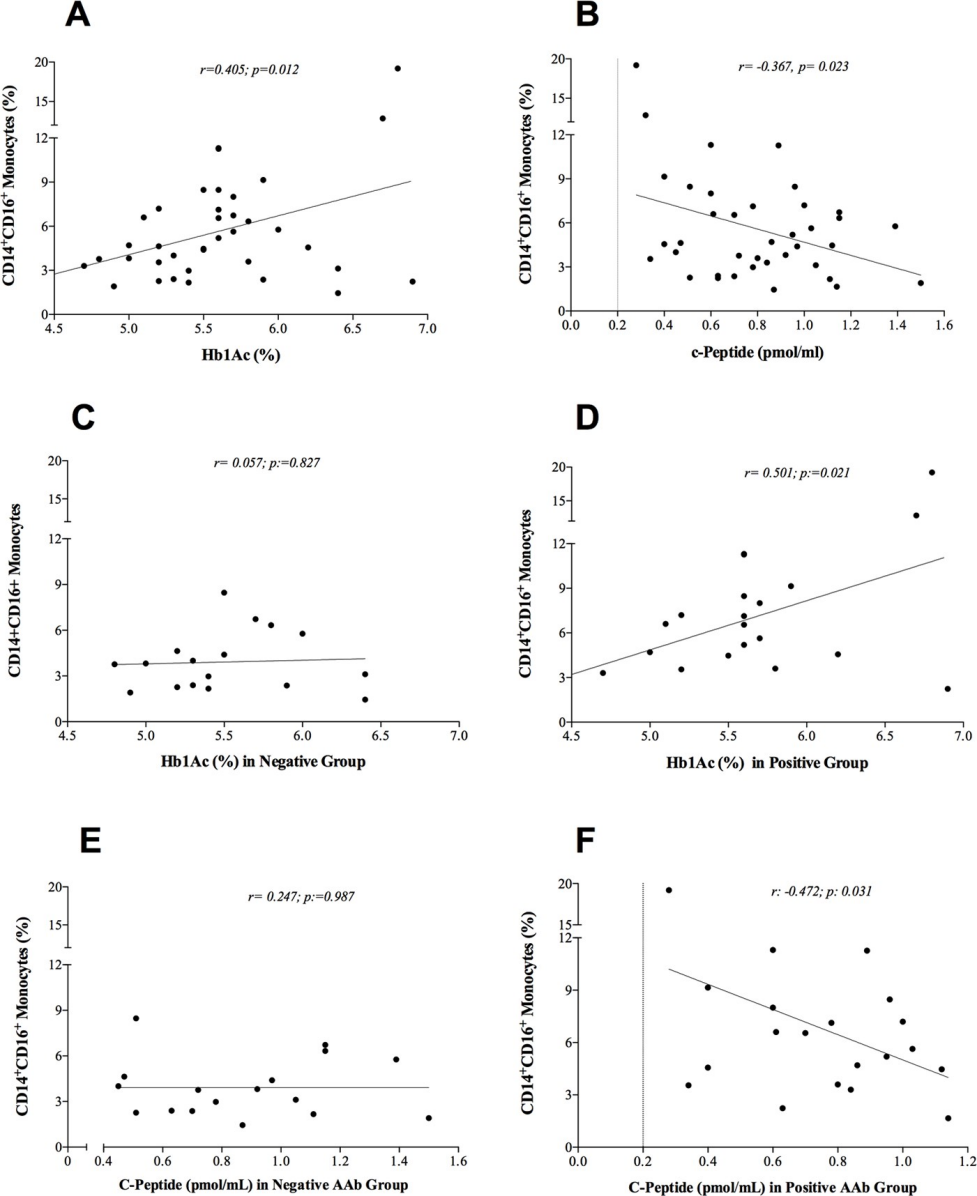
Plots showing the correlations between glycated hemoglobin, c-peptide serum levels, and monocytes. The continuous line represents the regression line. Correlation between the CD14^+^CD16^+^ monocytes and Hb1Ac levels **(A)** and c-Pep serum levels **(B).** Patients were stratified according to the positivity or negativity of pancreatic AAb. Among patients with negative AAb, Hb1Ac and C-Pep serum levels showed no correlation with the CD14^+^CD16^+^ monocytes **(C** and **E).** On the contrary, these correlations remained significant among those with positive AAb **(D** and **F)**.

Among parameters associated with pancreatic graft function, fasting glucose did not correlate with the CD14^+^CD16^+^ monocytes (Pearson correlation test; r = 0.209, p = 0.20). However, we found a positive correlation between CD14^+^CD16^+^ monocytes and Hb1Ac levels and an inverse correlation between the pro-inflammatory monocytes and the C-pep serum levels (Fig 3A and 3B). Patients were further stratified according to AAb status and separately performed the correlation tests. In AAb- patients there was no correlation between Hb1Ac and the C-pep and the CD14^+^CD16^+^ (Fig 3C and 3D), however, in AAb+ patients, the correlation between both parameters (Hb1Ac and C-pep) and the pro-inflammatory monocytes remained significant (Fig 3E and 3F).

### Interleukin 17A and Type II IFN-y

**Fig 4:**
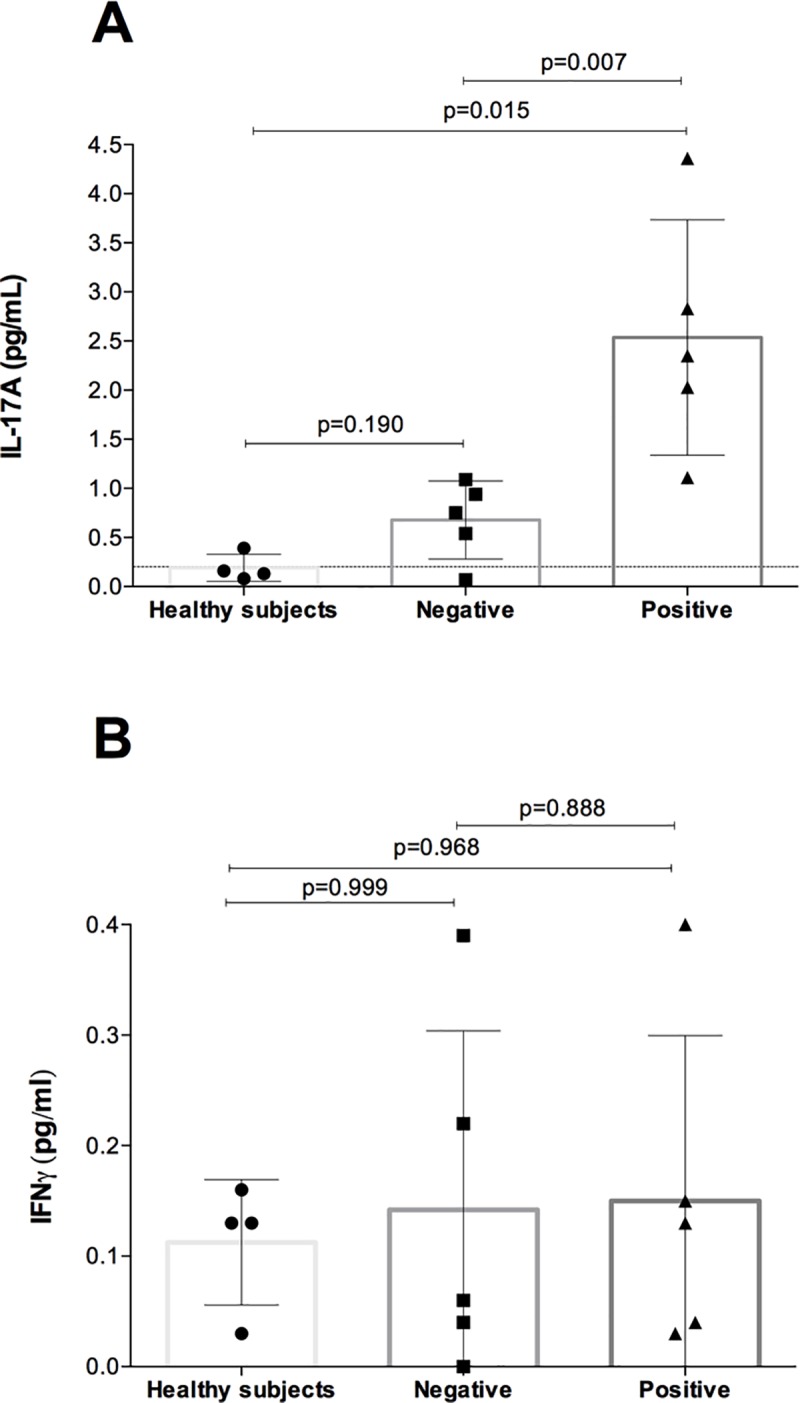
Plots showing IL-17A and IFN-*y* plasma values among five patients included. **(A)** Patients with positive AAb showed higher levels of IL17A compared to those with negative pancreatic AAb and the healthy controls. **(B)** Plot showing IFN*y* among different groups.

We also sought to evaluate whether there were any systemic signatures of the immune activation. Fig 4 shows the mean baseline serum levels of IL-17A and IFN*y*. AAb+ patients showed significantly increased serum levels of IL-17A compared to AAb- patients and the healthy controls (Fig 4A). IL-17 serum levels between the healthy control group and those with negative AAb were similar. Serum levels of IFN-*y* were comparable between the groups (Fig 4B).

### Logistic regression analysis

The univariate logistic regression analysis determined that the presence of AAb+ was associated with high CD14^+^CD16^+^ monocytes percentage (Non-adjusted OR 1.49, 95% CI 1.07–2.06, p = 0.017; Table 2). The parameters found to be significant in the univariate logistic regression analysis and those that may influence AAb positivity were then included in the multivariate analysis. After adjustment, the pro-inflammatory monocytes remained statistically significant in the multivariate logistic regression model (adjusted-OR 1.59, 95% CI 1.05–2.40, p = 0.027; Table 3). Interestingly, including mean time since transplantation in the multivariate model did not influence the results.

**Table 2:** Univariate logistic regression analysis showing the factors related to positivity or negativity for pancreatic autoantibodies after SPK.

Univariate Logistic regression model	OR	95% CI	p
**Age, (years, mean ± SD)** **Age, n (%)** ^**1**^ *<35>35*	0.98 1 0.93	0.88–1.08 0.25–3.41	0.70 0.91
**Gender***Female* *Male*	1 1.30	0.30–5.63	0.72
**BMI** ^**a**^ **(Kg/m**^**2**^**)**	0.96	0.82–1.11	0.60
**DR3/DR4 alleles, n (%)** *NoYes*	10.53	0.11–2.56	0.43
**Transplantation Vintage (months)** ^**b**^	0.99	0.97–1.01	0.38
**HLA Mismatch**	2.06	0.75–9.03	0.13
**Fasting Glucose** ^**(**^**mmol/ml)** ^**c**^	2.02	0.70–5.77	0.18
**Hb1Ac (%)** ^**d**^	1.55	0.47–5.16	0.46
**C-peptide (pmol/ml)**	0.16	0.01–1.71	0.13
**Albumin (g/l)**	1.26	0.18–8.77	0.81
**Ferritin (ng/mdl)**	1	0.99–1.0	0.92
***hs*-CRP** ^**e**^ **(mg/l)**	1.03	0.87–1.21	0.68
**GFR** ^**f**^ **(mil/min/1.73 m**^**2**^**)**	1.01	0.98–1.05	0.31
**Corticosteroids Dose (mg/day)**	0.97	0.69–1.35	0.85
**Tacrolimus levels** ^**g**^ **(ng/ml)**	1.11	0.77–1.61	0.56
**CD14**^**+**^**CD16**^**+**^ **Monocytes (%)**	1.49	1.07–2.06	0.017

^a^ BMI, Body Mass Index

^b^ Transplantation Vintage, Time Since Transplantation

^c^ Fasting Glucose, Serum fasting glucose

^d^ Hb1Ac, Glycated hemoglobin

^e^ CPR, C-reactive protein

^f^ GFR, Glomerular filtration rate

^g^ Tacrolimus trough serum levels.

^1^ Age-stratified as a binary variable (younger and older than 35)

**Table 3:** Multivariate logistic regression analysis to determine the adjusted model of the factors related to positivity o negativity of autoantibodies according to factors modulating the expression of pro-inflammatory monocytes.

Multivariate logistic regression model	Adjusted-OR	95% CI	p
**DR3/DR4 Allele No Yes**	Ref 0.89	0.13–5.91	0.90
**Transplantation Vintage (months)** ^**a**^	1.0	0.97–1.04	0.58
**Corticosteroids Dose (mg per day)**	1.21	0.70–2.11	0.48
**Tacrolimus Levels (ng/ml)** ^**b**^	1.05	0.67–1.65	0.82
**CD14**^**+**^**CD16**^**+**^ **Monocytes (%)**	1.59	1.05–2.40	0.027

^a^ Transplantation Vintage, Time Since Transplantation

^b^ Tacrolimus trough serum levels.

The multivariate model showed good calibration and discrimination ability. A receiver operator characteristics (ROC) curve analysis was performed to predict the increased monocytes percentage results based on the multivariate model. The area under the curve (AUC) was 0.75 (95% CI 0.60–0.91; p = 0.006). Positive predictive value (PPV) was 65.3% which means the ability of the model to predict the increased monocytes percentage in posttransplant AAb+ recipients (S2 Fig). On the other hand, negative predictive value (NPV) was 68.5%. Finally, the sensitivity of the model was 80.9%, expressing the percentage of patients correctly identified by the model. Specificity was 47.0%, indicating that only 43 out of 100 AAb- subjects were appropriately identified by the model.

## Discussion

In the present study, we aimed to evaluate the association between the pancreatic AAb positivity and glucose control in SPK recipients. We have shown that patients who were AAb+ after transplantation were more likely to have higher levels of Hb1Ac and lower C-pep levels after the first year of AAb positivity, suggesting an impairment of pancreatic ß-cells. Furthermore, AAb+ patients showed a higher proportion of CD14^+^CD16^+^ monocytes compared to those AAb- and the healthy control group. These pro-inflammatory monocytes showed a positive correlation with Hb1Ac serum levels and a persistent inverse correlation with C-pep levels among AAb+ patients. The higher percentage of pro-inflammatory CD14^+^CD16^+^ monocytes in peripheral blood in AAb+ patients compared to either AAb- and the healthy controls suggest an underlying micro-inflammatory status. Also, systemic signatures of immune activation were demonstrated by the fact that AAb+ patients had increased levels of IL-17A.

Recent studies have shown the association between pancreatic AAb and the development of T1D in children [20]. Reports on SPK recipients have described the selective loss of insulin secretion after SPK in AAb+ patients in the absence of pancreas graft rejection regardless of immunosuppressive therapy [25]. Such a study demonstrated that the presence of AAb after SPK was related to worse glucose control, insulitis, ß-cell loss on pancreas graft biopsy and circulating CD4-CD8 auto-reactive T-cells. Interestingly, AAb positivity preceded hyperglycemia by years, suggesting that the underlying mechanisms by which pancreatic AAb addresses ß-cell impairment overcome immunosuppressive medications [25]. A previous study evaluating the association between pancreatic AAb and the endocrine function of the pancreas graft failed to show any differences in neither fasting glucose, Hb1Ac, nor glucose tolerance test among SPK recipients with positive pancreatic AAb [30]. Our results are different from the prior study due to the observed progressive reduction of C-pep and the increase in Hb1Ac serum levels during follow-up in the group of patients evaluated longitudinally. The differences between studies might rely on the different methodological approach. While our results are based on a longitudinal setting, the study above was a cross-sectional analysis. So, the process is likely to be progressive and might be exclusively appreciated in the longitudinal setting as in our study. However, because of non-linear disease progression and the variable time for pancreatic AAb to become positive, the mechanisms preceding the activation of the immune process leading to the impairment of the ßeta-cell function regardless of immunosuppressive medications remain unknown. Therefore, as pancreatic autoantibodies are likely to impact the pancreas graft negatively after SPK [28,29], the identification of the mechanism preceding this clinically-evident autoimmune process is needed. In this line, evidence on pancreatic AAb to be the direct cause of ßeta-cell destruction or whether they act as whistleblowers of an ongoing underlying immunological event is limited. Some of the expressed autoantigens are recognized by some of the pancreatic AAb which shares target sites with self-reactive T-cells. Additionally, GAD-autoreactive CD4 T-cells have been observed in the circulation of patients as well as in local lymph nodes which suggests the activation of an immune process associated with pancreatic AAb [2]. Our study shows that the immune activation in AAb positive patients after SPK is not only a matter of T-lymphocytes but also includes monocytes. Activated monocytes induce IL-17 secreting cells from memory T-cells. Such cells are associated with the expansion of the immune process. Indeed, although the true mechanism remains a conundrum, in vitro studies have shown that activated monocytes and some of the chemokines secreted may induce endothelial senescence and T-CD4 cells expansion that eventually secretes lytic cytokines that may disrupt the endothelium allowing the T-cells to infiltrate different organs such as the pancreas. [31] Therefore, AAb may indirectly activate the immune process by promoting the activation of monocytes and CD4/CD8 T-cells. Monocytes are highly associated with TNF-α production which in turn expand T-lymphocytes secreting IL-17 cells [19]. Furthermore, activated monocytes may be recruited to the pancreatic islets [32]. In our study, the CD14^+^CD16^+^ monocytes showed a positive correlation with Hb1Ac levels and an inverse correlation with C-pep serum levels that remained significant after stratification only in AAb+ patients. Because C-pep serum levels are a robust way of measuring insulin production, these findings suggest an impairment of pancreatic ß-cell that could be associated with monocytes. Moreover, in multivariate logistic regression analysis, the raised percentage of pro-inflammatory monocytes was independently associated with being AAb+ after SPK despite immunosuppression and transplantation vintage. Further studies should examine whether pancreatic AAb positivity may promote and expand the stimulation of lymphocytes through the activation of pro-inflammatory monocytes that consequently induce ß-cell impairment and graft loss.

IL-17A regulates the trafficking of some immune cells including monocytes. In fact, an in-vitro study demonstrated that the blockade of IL-17A by an IL-17A mAb prevented the adhesion of monocytes [33]. Furthermore, IL-17 has also been associated with a detrimental effect on pancreatic islets. Indeed, neutralization of IL17 prevented the development of T1D in mice models [10]. A recent study, regarding the plasticity of Th1 and Th17 cells, showed that the gene expression of IL-17 and IFN-*y* were higher in patients with advanced impairment of beta-cell function and positive pancreatic AAb [10]. We found that those AAb+ patients showed higher levels of IL-17A compared with AAb- patients and the healthy controls. However, we did not observe any correlation between the IL-17A serum levels and neither the Hb1Ac nor the C-pep serum levels. Previous studies evaluating this correlation were conducted in non-transplanted patients that underwent an oral glucose tolerance test and formerly classified as impaired glucose tolerance [10]. In contrary, our study included patients considered to have a normal pancreas graft functioning and blood samples were drawn under fasting. Hence, our findings suggest that systemic signatures of inflammation may be present in patients with positive pancreatic AAb even during fasting in patients with normal graft function.

Moreover, like others [19], we failed to demonstrate any correlation between CD14^+^CD16^+^ monocytes and IFN-*y* serum levels. Recent studies have shown the association between pancreatic AAb and the increase in different cytokines such as IFN-*y* [34]. However, IF*N-y* may be increased only at later stages of pancreas dysfunction when T1D is diagnosed. In fact, it is likely that IFN-*y* secretion may be appropriately promoted only when glucose levels are high [10]. Hence, the variability of cytokines production over time, and the clinically within the normal range in C-pep and Hb1Ac serum levels may explain the lack of association in our study.

Regarding the effect of Immunosuppressive medications on monocytes, some interesting studies have reported that glucocorticoids are associated with apoptosis of monocytes [35,36]. Because all the patients included in our study were under a similar immunosuppressive therapy, we consider that whether or not these treatments played a role in the modulation and expression of the cytokines and monocytes subset analyzed, it must have affected all patients equally. Indeed, in our study, AAb- patients and the healthy controls showed a similar percentage of CD14^+^CD16^+^ monocytes which strengthen not only the ability of corticosteroids to modulate these monocytes subset but also of the pancreatic AAb to promote the expression of pro-inflammatory monocytes regardless of the corticosteroids. Furthermore, these facts are suggested by the comparable IFN*y* serum levels found in our patients. To date, no immunosuppressive protocols have demonstrated to prevent T1D development. However, recent research has suggested that the modulation of the Foxp3^+^ regulatory T-cells (Tregs) and the use of cytokine therapy with IL-2 (NCT02772679) may partially prevent ßeta-cell autoimmunity [37,38]. Nonetheless, whether these treatments may predispose to higher rates of infections and cancer remain to be elucidated. That being said, we consider that due to the remarkable role of T-cells in the development of T1D, its modulation could be the backbone for future investigation to prevent T1D development and recurrence.

CD14^+^CD16^+^ monocytes subpopulation has been previously investigated in other transplant population [36], but not on SPK recipients. To the best of our knowledge, this is the first study that analyzes and correlates the presence of pancreatic AAb with CD14^+^CD16^+^ monocytes after SPK. Surprisingly, no association between AAb and peripheral pro-inflammatory monocytes has been found or whether these monocytes are linked to the autoimmunity process leading the impairment of ß-cell of pancreas graft after SPK so far.

Our study has some limitations. First, we did not consider pretransplant pancreatic AAb serostatus. However, recent evidence has suggested that patients who seroconverted after SPK are those in the higher risk of type 1 diabetes recurrence [28], meaning that pre-transplant autoantibody status could not have any influence in a worse pancreas endocrine function after SPK. Second, due to the restrictive inclusion and exclusion criteria to include a much possible homogenize sample, a relatively small subset of patients was included in the cross-sectional study. Third, blood samples were drawn under fasting, and some of the negative results could be underestimated. Our results are based on fasting serum levels of Hb1Ac and c-peptide, and the determination of such parameter under fasting in contrast to the dynamic tests may reflect different information about the ß-cell functioning. We consider that either the oral glucose tolerance test or the mixed meal challenge could be a reasonable option for those patients with positive AAb with previous glucose impairment. Hence, further research should address dynamic tests in SPK recipients. Fourth, we did not assess the effect that some other medication such as angiotensin II receptor AT1 antagonists may have on the results as they have shown the ability to modulate the pro-inflammatory monocytes in other population [39]. Fifth, we measured the circulating levels of IL-17A. However, we failed to estimate the total and the percent number of Th-17 cells. Former studies addressing such a subject have indeed reported that the number and the ability of such cells to produce higher amounts of IL-17 is increased at the time of T1D diagnosis [10]. In fact, the IL-17 pathway is upregulated in those children with impaired glucose tolerance as well as those recently diagnose of T1D. However, no evidence of activated circulating Th-17 secreting cells was seen in the early stages of immune activation. Since the patients included in the cross-sectional cohort of our study were considered to have a good pancreas graft function at the time of inclusion, the Th-17 circulating cells were not evaluated. Further studies should be performed to evaluate whether these inflammation-related cells profile are also increased in SPK recipients regardless of the good pancreas performance. Finally, although AAb were assessed prospectively in the longitudinal study, the cross-sectional part of this study included isolated blood drawn, and their presence may randomly vary over time. Conversely, all monocytes and cytokines measurements for each assay were performed in duplicate in the same laboratory to minimize other potential bias.

## Conclusions

Posttransplant pancreatic autoantibodies were associated with a progressive increase in Hb1Ac and a decrease in C-pep serum levels after SPK. Also, pancreatic AAb positivity was independently associated with an increased percentage of CD14^+^CD16^+^ monocytes and IL-17A serum levels. Despite the immunosuppressive therapy, SPK recipients with positive AAb showed signs of immune activation at different levels. However, it might not accurately reflect the presence of a settled autoimmune process over the pancreatic graft. Even though we cannot discern true mechanism from this study, it could be considered hypothesis generating. Peripheral blood biomarkers of the disease that correlate with clinical finding of glucose impairment lack. In this line, monocytes have been demonstrated to lead the differentiation and expansion of CD4 cells into Th17/Th1 cells. Hence, linking the pancreatic AAb and the pro-inflammatory monocytes points inflammation as a possible factor affecting pancreas function. Clinically, recognizing the very early different immune cells involved in the impairment of the ßeta-cell function may have an impact on the development of new strategies regarding immunosuppressive protocols for SPK recipients with positive AAb. Nevertheless, further prospective longitudinal studies should evaluate whether the modulation of these pro-inflammatory monocytes could improve the underlying micro-inflammation and thus the pancreas graft function as well as their impact on recipients’ survival.

## Supporting information

S1 Dataset Compilation of data from patients included in the longitudinal analysis. (CSV)

S2 Dataset Compilation of data from patients included in the cross-sectional analysis. (CSV)

S1 Fig Glycated hemoglobin and C-peptide serum levels in the group of patients prospectively followed-up. Patients with positive AAb showed higher levels of Hb1Ac in parallel with lower c-Pep serum levels along time regardless of the subtype of AAb. Differences were different after the first year following seroconversion. *p<0.05. ^&^p>0.05. All comparisons are with the AAb negative group.
(DOC)

S2 Fig ROC curve comparing patients with positive autoantibodies and negative autoantibodies. The AUC was 0.75 (95% CI 0.60–0.91; p = 0.006), PPV was 65.3% and NPV was 68.5%. The sensitivity of the model was 80.9%, and specificity was 47.0%.
(DOC)
