# Supplementary material for: Pancreatic autoantibodies and CD14+CD16+ monocytes subset are associated with the impairment of ß-cell function after simultaneous pancreas-kidney transplantation
Source: PLoS One. 2019 Feb 22;14(2):e0212547. doi: 10.1371/journal.pone.0212547 (PMC6386378; doi:10.1371/journal.pone.0212547)

**Supplemental Information**

**S1 Fig.** **Glycated hemoglobin and C-peptide serum levels in the group of patients prospectively followed-up**. Patients with positive AAb showed higher levels of Hb1Ac in parallel with lower c-Pep serum levels along time regardless of the subtype of AAb. Differences were different after the first year following seroconversion. *p<0.05. ^&^p>0.05. All comparisons are with the AAb negative group.


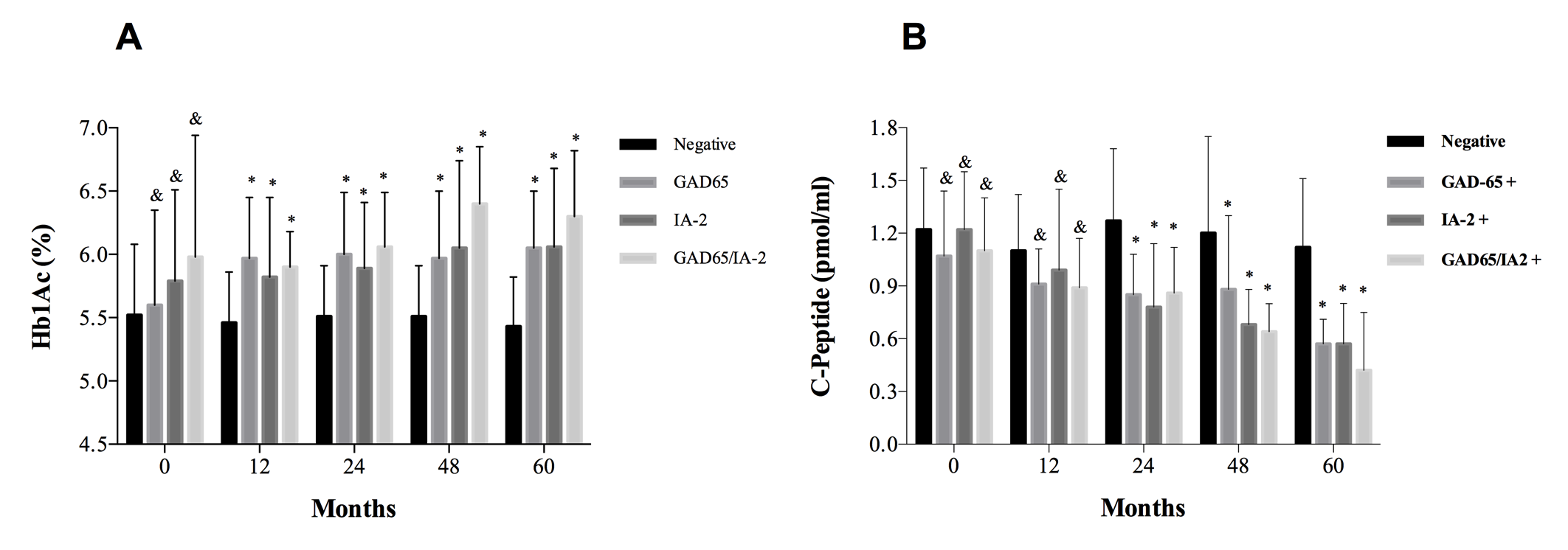

Supplement: S1 Fig — Patients with positive AAb showed higher levels of Hb1Ac in parallel with lower c-Pep serum levels along time regardless of the subtype of AAb. Differences were different after the first year following seroconversion. *p<0.05. &p>0.05. All comparisons are with the AAb negative group. (DOC) [file pone.0212547.s003.doc]
