# Supplementary material for: Pancreatic autoantibodies and CD14+CD16+ monocytes subset are associated with the impairment of ß-cell function after simultaneous pancreas-kidney transplantation
Source: PLoS One. 2019 Feb 22;14(2):e0212547. doi: 10.1371/journal.pone.0212547 (PMC6386378; doi:10.1371/journal.pone.0212547)

**Supplemental Information**

**S2 Fig**. **ROC curve comparing patients with positive autoantibodies and negative autoantibodies.** The AUC of the model was 0.75 (95% CI 0.60-0.91; p=0.006), PPV was 65.3% and NPV was 68.5%. Sensitivity of the model was 80.9% and specificity was 47.0%.


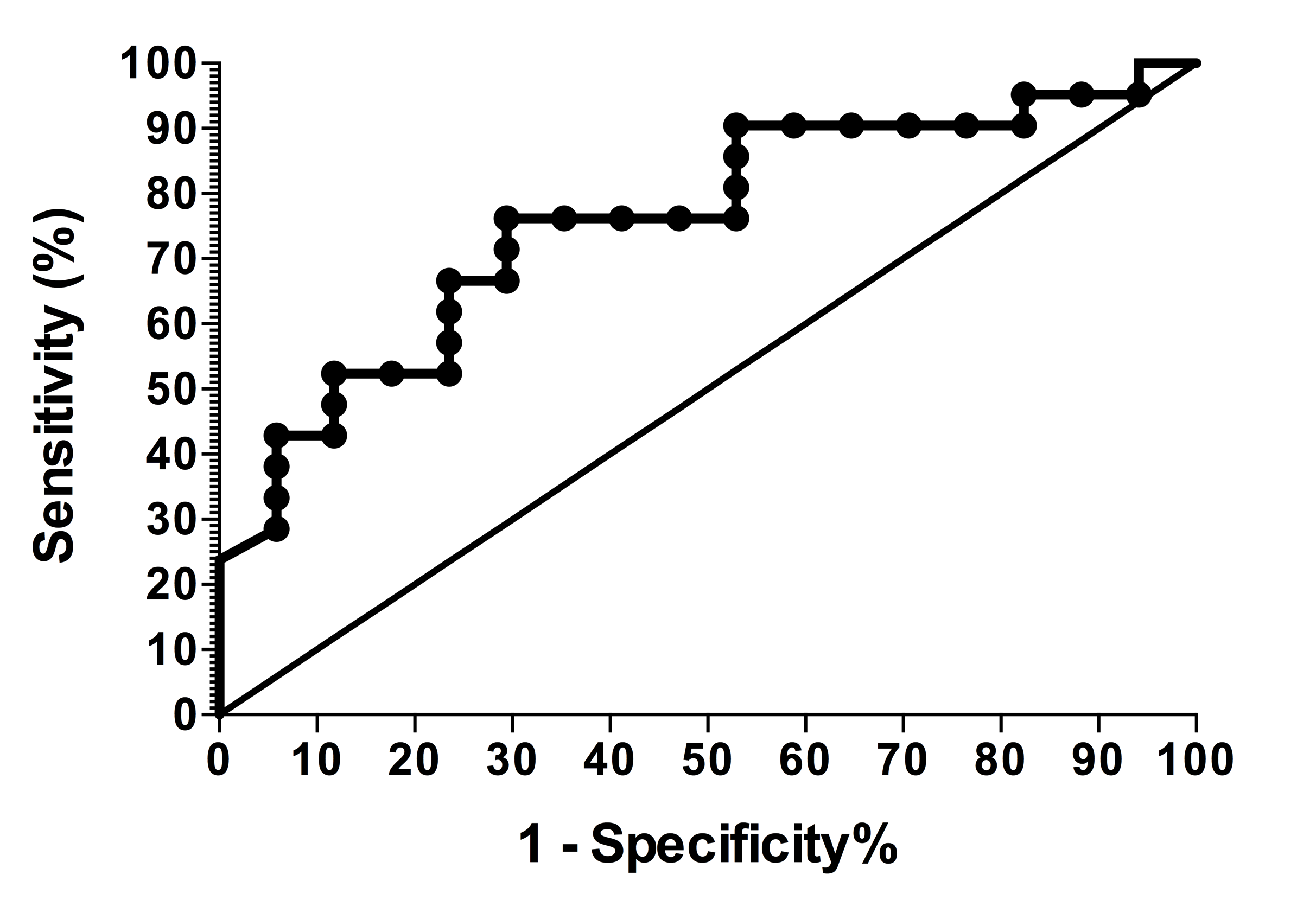

Supplement: S2 Fig — The AUC was 0.75 (95% CI 0.60–0.91; p = 0.006), PPV was 65.3% and NPV was 68.5%. The sensitivity of the model was 80.9%, and specificity was 47.0%. (DOC) [file pone.0212547.s004.doc]
